# Genome-Wide Association Studies of Myocardial Infarction: A Systematic Literature Review

**DOI:** 10.3390/jcdd13030127

**Published:** 2026-03-10

**Authors:** Isabelle P. Thierry, Reza Jabbari, Thomas Engstrøm, Jacob Tfelt-Hansen, Charlotte Glinge

**Affiliations:** 1 The Heart Centre, Department of Cardiology, Copenhagen University Hospital, Rigshospitalet, DK 2100 Copenhagen, Denmark; 2 Department of Forensic Medicine, Faculty of Medical Sciences, University of Copenhagen, DK 2100 Copenhagen, Denmark

**Keywords:** genome-wide association study, genetics, myocardial infarction, single nucleotide polymorphisms, polygenic risk score, review

## Abstract

Myocardial infarction (MI) remains a leading cause of morbidity and mortality worldwide, which can result in severe complications such as cardiac arrhythmia, heart failure, and sudden cardiac death. Genetic factors contribute to MI etiology and have been studied through genome-wide association studies (GWAS). This systematic review aims to summarize all GWAS of MI reporting single-nucleotide polymorphisms (SNPs) reaching genome-wide significance (*p* < 5 × 10^−8^) and elucidate on their biological relevance and potential clinical utility. A systematic review following PRISMA guidelines was conducted using PubMed and the GWAS Catalog to identify eligible studies. This review included nine GWAS published between 2007 and 2023, conducted in both European and non-European cohorts. GWAS have identified multiple loci associated with MI, pinpointing potential biological pathways underlying MI, and potential therapeutic targets and enhancing risk prediction. Nonetheless, significant challenges remain, particularly the underrepresentation of diverse ancestries and the need for functional follow-up studies to define causal variants and clarify the mechanisms linking genetic variation to MI pathogenesis.

## 1. Introduction

Myocardial infarction (MI) remains a worldwide challenge for health and social care systems, since it is a leading cause of death with severe complications such as cardiac arrhythmia, heart failure, and sudden cardiac death [1,2,3,4]. The development of MI is influenced by both environmental and genetic factors. MI can present as early-onset, which is often indicative of an underlying genetic predisposition [5,6]. Traditional risk factors such as smoking, hypertension, and diabetes have long been established risk factors for MI [4]. Genetic predisposition to MI was first implicated after family and twin studies observed aggregation of MI within families [7,8]. Initially, MI was considered a monogenic disorder caused by rare variants in genes such as *LDLR*, *APOB*, and *PCSK9*, causing familial hypercholesterolemia [9]. The genetic architecture of MI is more complex than initially thought. Although common variants identified through genome-wide association studies (GWAS) account for an estimated 30–40% of MI heritability, they explain only a fraction of the overall genetic contribution [10,11]. The remaining “missing heritability” likely resides in rare variants of larger effect, gene–environment interactions, structural variation, and epigenetic modifications that are not fully captured by standard GWAS approaches [12]. It could be speculated that MI follows an omnigenic model, in which all genes expressed in relevant tissues may contribute to the pathophysiology [13]. To elucidate this omnigenic model of MI, genome-wide association studies (GWAS) have become essential. GWAS examine the genome in a hypothesis-free manner to identify common variants associated with MI [14]. MI was one of the earliest and most comprehensively studied phenotypes through GWAS approaches [15].

**Figure 1:**
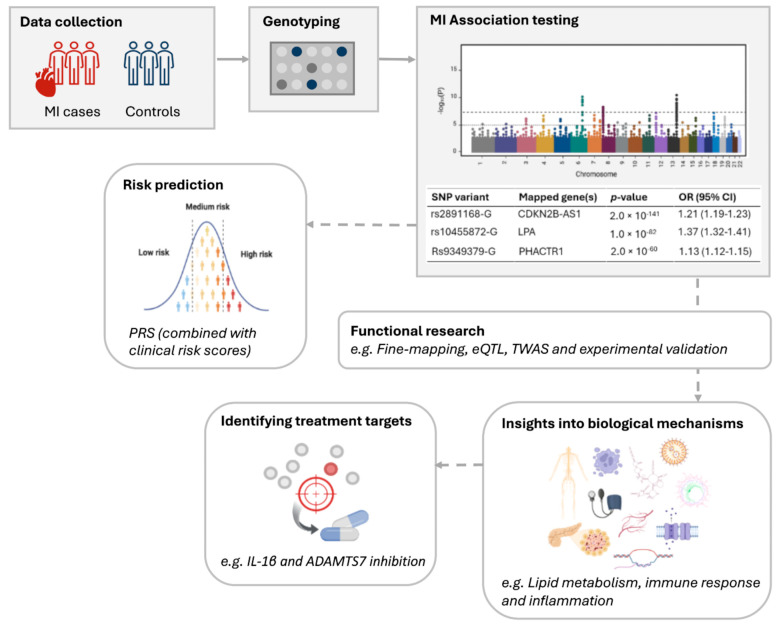
Overview of GWAS of MI workflow and its clinical implications. The GWAS workflow is shown in the gray boxes. The clinical implications of the GWAS results are shown in the white boxes. Genetic data are obtained for individuals with and without an MI phenotype. Genotyping of the genomes of the included individuals is performed. GWAS results are visualized in a Manhattan plot, with the y-axis representing −log10 *p*-values and the x-axis representing chromosomal positions. The effect sizes of associated SNPs are used for risk prediction with polygenic risk scores, which can be combined with clinical risk scores using non-genetic risk factors. The SNP variants associated with MI can be further analyzed by performing functional research. This can provide insights into biological mechanisms associated with MI and pinpoint potential treatment targets. EQTL, Expression quantitative trait locus; GWAS, Genome-wide association study; MI, Myocardial infarction; PRS, Polygenic risk score; TWAS, Transcriptome-wide association studies [14,20,21,22,23,24,25].

GWAS compare the allele frequency of common variants, or single-nucleotide polymorphisms (SNP), across the entire genome between groups of individuals with shared ancestry, usually a group with a particular disease or trait (cases) and a control group without it. For example, MI cases and controls without MI (Figure 1) [14]. Shared ancestry is essential, as allele frequencies vary across populations with different ancestral backgrounds [16]. After comparing allele frequencies between MI cases and controls, SNPs that occur significantly more frequently in MI cases are considered associated with MI. Given the large number of SNPs across the genome, multiple testing correction is performed [17]. Therefore, an SNP is considered genome-wide significant when it has a *p*-value of *p* < 5 × 10^−8^. These genome-wide significant SNPs pinpoint loci associated with MI. A locus (plural: loci) is a topological reference point referring to a region on the chromosome. These genomic loci can span up to hundreds of kilobases, containing coding regions, encoding for proteins, and non-coding regions that regulate gene expression [18,19]. Notably, the majority of GWAS-identified SNPs are located in non-coding regions, highlighting the importance of regulatory elements in disease susceptibility.

An SNP significantly associated with MI is not necessarily a causal variant, as SNPs can be in linkage disequilibrium (LD) with each other. This means that these SNPs are statistically associated and tend to be inherited together in a population because of their physical proximity on the chromosome [26]. Therefore, an SNP identified with GWAS may not be causal itself but could be in LD with the causal variant. Determining what variant is causal requires functional research. First, fine-mapping pinpoints the likely causal variants among SNPs in LD [20], followed by functional annotation to determine the biological relevance of the variants [21]. Variants are then linked to target genes using approaches such as expression quantitative trait locus (eQTL) mapping and transcriptome-wide association studies (TWAS) [22]. Finally, these genes can be connected to molecular pathways, and experimental validation can be performed [23].

Insights into the genes and molecular pathways underlying MI can have a clinical impact by identifying potential treatment targets and improving personalized risk prediction of MI in individuals [23]. Polygenic risk scores (PRS) are generated to identify individuals at elevated risk for MI. It enables the identification of those individuals even before clinical symptoms appear [24]. This offers opportunities for early interventions, personalized prevention strategies, and refined risk stratification beyond traditional factors. A PRS is calculated using the effect size of SNPs identified in GWAS [27].

This systematic review aims to summarize all GWAS of MI reporting single-nucleotide polymorphisms (SNPs) reaching genome-wide significance (*p* < 5 × 10^−8^) and elucidate on their biological relevance and potential clinical utility.

## 2. Materials and Methods

This systematic review was conducted following the Preferred Reporting Items for Systematic Reviews and Meta-Analyses (PRISMA) guidelines (see Supplementary Materials) and was registered in the Open Science Framework (OSF) (Registration number: nztkh) [28].

Studies were included in this systematic review when (1) a GWAS of MI was performed, defined as a GWAS comparing MI cases to controls without MI, and (2) the identified SNPs were genome-wide significant (*p* < 5 × 10^−8^). Two reviewers independently performed the search for eligible studies in the PubMed database and the GWAS Catalog, which was last updated on 1 October 2025.

The GWAS Catalog is the online catalog of GWAS hosted by the National Human Genome Research Institute. The catalog was established to provide a single reliable and freely available database of SNPs associated with specific traits and diseases. Studies are included in the GWAS Catalog when (1) it analyses the human genome without limiting to candidate genes, (2) includes a primary GWAS analysis (defined as “array-based genotyping and analysis of 100,000+ pre-QC SNPs selected to tag variation across the genome and without regard to gene content”), and (3) was published in English [29].

The SNPs reported in this review are the genome-wide significant SNPs reported by the GWAS Catalog. The GWAS Catalog reported the SNPs in genome construct hg38 as follows: When a combined *p*-value was not reported, the *p*-value and effect size from the largest sample size were reported (as long as the samples showed *p* < 1 × 10^−5^ in initial and replication samples). When no replication was performed, the SNPs identified in the discovery stage were reported. When the SNP was the most significant SNP from an independent locus and when previously known SNPs (at the time of publication) met the genome-wide significance threshold, even without replication, they were reported.

## 3. Results

**Figure 2:**
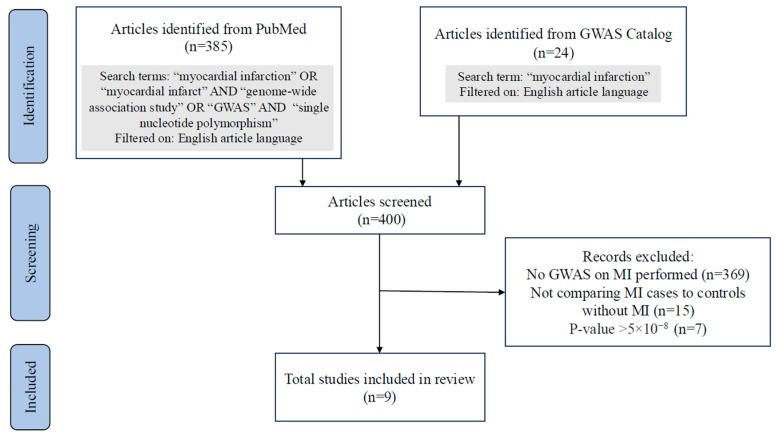
PRISMA Flowchart of article search for GWAS of MI. Flowchart representing the search for GWAS on MI performed in PubMed and the GWAS Catalog on 1 October 2025. GWAS, Genome-wide association studies; MI, Myocardial infarction.

The search for MI GWAS yielded nine eligible studies published in a period of 17 years (from 2007 to 2023) (Figure 2 and Table 1). Five European studies and four non-European studies were performed. The study characteristics are shown in Table 1. Together, the studies identified 198 genome-wide significant SNPs on independent loci, including 110 reported in European and 144 in non-European cohorts (Tables S1 and S2).

In 2007, the first GWAS of MI in European cohorts that identified a genome-wide significant SNP was performed [30]. They identified the common variant rs10757278 on chromosome 9p21, which contains the sequence encoding for CDKN2A/B, a tumor-suppressor gene regulating cell growth and division, whose association with MI was unknown before [37]. Currently, this locus, 9p21.3, is the most replicated associated locus with MI (Table S1). Subsequent studies in European cohorts identified more genome-wide significant variants associated with MI [5,31,33], including a variant on the *ABO* locus [31], encoding for proteins related to the blood group system [38], and the variant rs6941513 near *QKI*, encoding for a protein that is a regulator of pre-mRNA alternative splicing, which is highly expressed in adult and developing hearts [39]. Multiple variants identified in these GWAS were previously identified as variants associated with coronary artery disease (CAD), the primary underlying condition leading to MI, which explains the observed overlap in variants associated with CAD and MI [40].

In recent years, consortia have been formed, such as Coronary Artery Disease Genetics Consortium (C4D), Coronary Artery Disease Genome-Wide Replication and Meta-Analysis (CARDIoGRAM) Consortium (merged in 2013 to CARDIoGRAMplusC4D) and the Myocardial Infarction Genetics Consortium, and large biobanks have been established [41,42,43]. This enabled the study of bigger cohorts, which increased the statistical power of GWAS. For example, the UK Biobank is an advanced biobank consisting of data on over 500,000 individuals in the UK and is available through its public website [44]. The most recently published GWAS of MI in European cohorts used this biobank, resulting in a cohort of 17,505 cases and 454,212 controls. The study identified 1966 SNPs on 31 loci with significant genome-wide association with MI. They replicated known variants for MI, on 9p21, and loci encoding for LPA and SORT1, and they identified eight new loci with genome-wide significance for MI. They subsequently confirmed the association of a locus on chromosome 1p21.3 harboring *SLC44A3* with MI in the Biobank Japan [35].

In 2011, the first GWAS of MI in non-European cohorts that identified a genome-wide significant SNP was performed. They identified a genome-wide significant variant on chromosome 5p15.3 that acts as a protective factor for MI [25]. Subsequent studies in non-European cohorts identified additional variants and loci associated with MI. Many of these overlapped with loci previously reported in European populations, including regions harboring *LPA*, *PHACTR1*, *CELSR2*, as well as the most frequently replicated locus 9p21 [35]. However, some identified variants and loci were only identified in European cohorts, for example, the locus harboring *APOE* [35], or only in non-European cohorts, for example, the locus harboring *ALDH2* [36]. This underscores the importance of conducting GWAS of cohorts representing diverse ancestries. To date, GWAS of MI primarily focused on populations from European, middle Eastern, and Asian ancestry. Performing GWAS of cohorts from other ancestries will further maximize gene discovery and reduce the health disparities between European and non-European populations [45].

### 3.1. Associated Pathways and Intermediate Phenotypes

**Figure 3:**
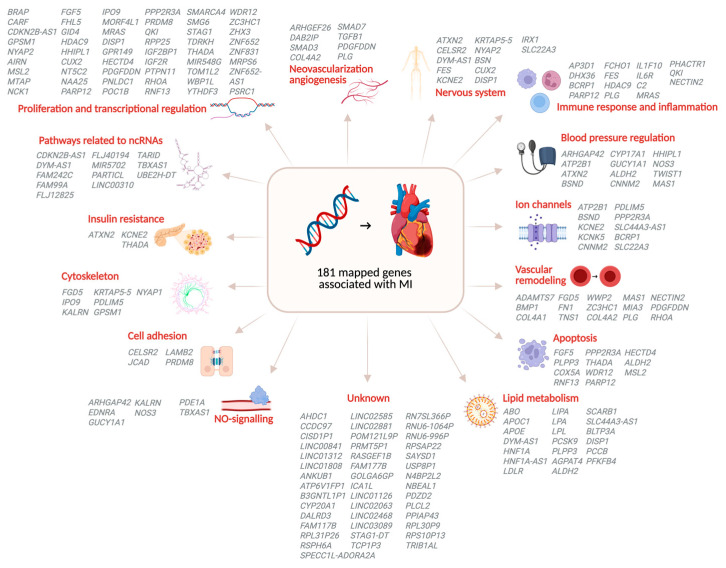
MI-associated genes and related pathophysiologic pathways and mechanisms. The GWAS of MI reported or mapped 181 genes. The pathways or systems the genes play a role in are shown in red. MI, Myocardial infarction (Figure concept inspired by Erdmann et al. [10,49]).

Multiple pathophysiologic mechanisms contribute to the development of atherosclerosis and ultimately MI, including dysregulation of lipid metabolism, diabetes, hypertension, and chronic inflammation [46]. GWAS of MI have clarified this complexity by identifying MI-associated loci enriched for genes governing metabolic, inflammatory, and vascular pathways (Figure 3) [46].

Atherosclerosis is initiated by endothelial dysfunction, which facilitates the accumulation of lipids such as low-density lipoprotein (LDL) within the arterial wall. This lipid deposition triggers an inflammatory response that further exacerbates endothelial dysfunction and lipid dysregulation, thereby promoting the progression, destabilization, and eventual rupture of atherosclerotic plaques [47]. GWAS of MI have identified multiple loci harboring genes involved in lipid metabolism, including *ABO*, *APOE*, *LDLR*, and *LPA* [35], as well as genes related to immune response and inflammation, such as *FES*, *IL6R*, and C2.

Moreover, diabetes and hypertension further accelerate plaque development and thrombosis. Diabetes promotes endothelial dysfunction through chronic hyperglycaemia, oxidative stress, and low-grade inflammation. Hypertension contributes via mechanical stress on the vessel wall, increased oxidative stress, and activation of inflammasome pathways, thereby enhancing the production of pro-inflammatory mediators [47]. Consistently, GWAS of MI have identified loci containing genes associated with diabetes, including *ATXN2* and *THADA*, and with hypertension such as *ARHGAP42*, *ATP2B1*, and *NOS3*.

Collectively, these interrelated processes increase susceptibility to MI. Notably, many genes mapped to MI-associated GWAS loci are implicated in these upstream cardiometabolic and inflammatory pathways, suggesting that most identified variants influence disease predisposition through modulation of chronic pathophysiologic mechanisms rather than through direct involvement in the acute infarction event itself.

These pathophysiologic mechanisms contributing to the development of MI influence quantifiable biological traits, often referred to as intermediate phenotypes, which serve as a mechanistic bridge between genetic variation and MI risk. Studying intermediate phenotypes, and the overlap between MI-associated variants and variants associated with these traits, may provide a more direct understanding of the biological processes underlying MI, early-stage MI, and atherosclerosis progression [48].

These findings illustrate that MI can be seen as a complex network of genetically influenced intermediate phenotypes, which must be understood to translate GWAS findings into biological mechanisms.

### 3.2. Loci in Coding and Non-Coding Regions

GWAS have identified MI-associated loci spanning both coding and non-coding regions, underscoring how variants in sequences encoding for proteins and regulating gene expression collectively shape MI susceptibility. The genes mapped to these variants are shown in Tables S1 and S2.

An example of an MI-associated loci in a non-coding region, is the most replicated locus, 9p21, that is harboring *CDKN2B-AS1*. *CDKN2B-AS1* is translated into long non-coding RNAs (lncRNA) that silence other genes in the *CDKN2B-CDKN2A* cluster, thereby regulating the gene expression of the cluster [37]. At first, the relation of this potential mechanism to the MI mechanism was unknown. By performing a functional study, Li et al. found that the CDKN2B-AS1 lncRNA inhibits *ADAM10* transcription, which promotes the efflux of cholesterol and prevents the atherosclerotic inflammatory response when active. Therefore, CDKN2B-AS1 can prevent atherosclerotic formation, a well-known cause of MI [40]. This demonstrates how functional studies can elucidate the role of a previously unknown mechanisms to MI. Furthermore, a study by Bayoglu et al. found that loci encoding for *CDKN2B-AS1* are associated with increased susceptibility to hypertension development [50]. Showing that CDKN2B-AS1 may be involved in multiple mechanisms contributing to MI.

An example of an SNP in coding regions is the SNP rs368803408 on a locus harboring the sequence for the low-density lipoprotein receptor (LDLR). Variants in *LDLR*, which mediates the uptake of cholesterol, are known to cause monogenic familial hypercholesterolemia, which is strongly associated with MI [9].

Life threatening arrhythmias may occur in the setting of acute MI [51]. It is thought that myocardial ischemia and subsequent scar formation are the primary mechanisms underlying arrhythmogenesis following acute MI [52]. However, not all MI patients develop arrhythmias. Factors such as infarct location, patient age and comorbidities are thought to influence this [53]. Additionally genetic factors may play a role in arrhythmogenesis following MI as well. For example, three SNPs rs9982601, rs928758, and rs28451064, identified in both European and non-European cohorts are located on the locus that harbors the sequence encoding for *KCNE2*. *KCNE2* is a voltage-gated potassium channel gene expressed in muscles and the heart that regulates, among others, smooth muscle contraction, heart rate, neurotransmitter release, and insulin secretion [54]. Loss-of-function *KCNE2* variants are associated with long-QT syndrome and arrhythmia, including atrial fibrillation [55]. These three SNPs, together with other ion-channel-encoding genes (Figure 3), point to specific pathways that may contribute to proarrhythmic susceptibility in MI. The fact that these variants are identified in GWAS of MI could be explained by the possibility that *KCNE2* variants may not only influence the severity of arrhythmias that arise secondary to MI but also suggest an underlying predisposition to both arrhythmias and MI itself. This highlights the need for further fundamental research to substantiate these findings.

### 3.3. Treatment Targets

GWAS of MI, in combination with, amongst others, Mendelian randomisation, have identified MI-associated mechanisms that were already targeted by established treatments, such as Proprotein convertase subtilisin/kexin type 9 (PCSK9) inhibitors and statins, as well as potential novel treatment targets [56,57].

PCSK9 regulates lipid homeostasis through binding to the low-density lipoprotein (LDL)-receptor and targeting it for degradation, resulting in an increased LDL-cholesterol concentration in the circulation [58,59,60]. High levels of PCSK9 are significantly associated with increased risk of cardiovascular disease [61,62]. Inhibition of PCSK9 has shown to reduce LDL-cholesterol by 60% [63], and can reduce risk of MI [64].

The first example of a treatment target pinpointed by GWAS results is interleukin-6 (IL-6). GWAS have identified variants at the IL6R locus associated with MI [35,36]. Inhibition of the IL-6 receptor (IL-6R) is suggested to have cardiovascular benefit. The CANTOS trial showed that inhibition of interleukin 1-beta (IL-1β), a component in the IL-6 signaling pathway, with a monoclonal antibody reduces cardiovascular event rates in patients with a history of coronary heart disease [65]. Early-phase trials of Tocilizumab, an IL-6R inhibitor originally approved for rheumatoid arthritis, have shown promising results for cardiovascular effects [66].

Another potential target identified by GWAS is *ADAMTS7* [36], which encodes a metalloprotease involved in extracellular matrix degradation and atherosclerosis progression [31]. Its causal role in atherosclerosis has been confirmed in vivo [67], and inhibitors of ADAMTS7 are currently being explored in preclinical studies [68,69].

### 3.4. Polygenic Risk Score for Early Risk Prediction of MI

PRS are generated to estimate an individual’s risk for MI, based on the MI-associated variants in the genome of the individual. A PRS is calculated as the sum of the weighted effect size of SNPs associated with MI, regardless of their individual genome-wide significance [27]. Therefore, a PRS captures the polygenic architecture of complex and common traits, such as MI, more comprehensively than a genetic risk score (GRS). A GRS is typically calculated by the sum of the effect size of the most significant variants, so not all identified SNPs [70]. As the predictive power of PRS improves with the identification of additional SNPs of larger effect sizes, its integration into clinical practice could advance precision medicine. GWAS of MI have identified SNPs with effect sizes up to 1.39 (95% CI, 1.33–1.44) (Tables S1 and S2).

Several studies have evaluated PRS in MI and CAD cohorts. Khera et al. showed the potential of a genome-wide PRS (comprising 6.6 million SNPs) for improving CAD risk prediction [24]. They identified 8% of the cohort at greater than 3-fold increased risk for CAD. This PRS was derived from a GWAS with 184,305 participants and was validated in the UK Biobank [44,71].

### 3.5. Combining Polygenic Risk Score with Clinical Risk Scores

Currently used clinical risk scores for recurrence of MI, such as GRACE (Global Registry of Acute Coronary Events) and TIMI (Thrombolysis in Myocardial Infarction) Risk scores, use commonly available clinical and laboratory variables that do not include genetic factors [72]. To improve risk prediction for MI, PRS can be combined with these clinical risk models. A study on PRS for cardiovascular diseases showed that combining PRS with clinical risk models significantly improved the identification of individuals who experienced a major adverse cardiovascular event. This increase was especially seen in younger individuals [73]. Adding PRS to standard clinical risk models of MI could therefore improve risk prediction for MI.

### 3.6. Limitations

Besides the limitations discussed throughout the chapters, it is important to highlight additional factors in GWAS of MI that could affect interpretation of GWAS results.

Not accounting for non-genetic risk factors, such as smoking, drug and alcohol abuse, in GWAS of MI may introduce confounding, as these environmental factors can increase MI risk and alter gene expression [74,75]. Therefore, observed genetic associations may represent gene–environment interactions, where genetic effects on MI risk are modified by environmental factors. For example, a genetic variant may only be associated with MI in individuals who smoke [76,77].

The definition of MI and the selection of cases and controls influence the studied phenotype and, consequently, GWAS results. The definition of MI differed between studies, ranging from diagnosis through ECG manifestations and changes in biomarkers [34] to self-reported MI (Table 1) [35]. Therefore, it is important to recognize that, although all included GWAS-identified variants associated with MI, some variants may reflect susceptibility to specific MI phenotypes captured in each study.

## 4. Conclusions

Over the past two decades, nine GWAS have identified genome-wide significant loci associated with MI, pinpointing potential biological pathways underlying MI and potential therapeutic targets and improving genetic risk prediction. Therefore, identification of MI-associated loci through GWAS of MI has led toward pathway-level understanding of MI. Nonetheless, significant challenges remain, particularly the underrepresentation of diverse ancestries and the need for functional follow-up studies to define causal variants and clarify the mechanisms linking genetic variation to MI pathogenesis.

## Figures and Tables

**Table 1:** Characteristics of GWAS of MI included in the systematic review.

Title	PMID	Author	Journal	Year	Study Cohort	Cases and Controls Definition
A common variant on chromosome 9p21 affects risk of myocardial infarction	17478679	Helgadottir A. et al.	Science	2007	1607 European ancestry cases, 6758 European ancestry controls	Cases with MI with age at onset <70 in males and <75 in females. Controls without history of CAD
Genome-wide association of early-onset myocardial infarction with single nucleotide polymorphisms and copy number variants	19198609	Kathiresan S. et al.	Nat Genet	2009	2967 European ancestry cases, 3075 European ancestry controls	Cases with MI with age at onset ≤50 in males and ≤60 in females. Controls without MI matched on sex and age
Identification of ADAMTS7 as a novel locus for coronary atherosclerosis and association of ABO with myocardial infarction in the presence of coronary atherosclerosis: two genome-wide association studies	21239051	Reilly MP et al.	Lancet	2011	5783 European ancestry cases, 3644 European ancestry controls	Cases undergoing cardiac catheterisation who had angiographic CAD and MI (≤55 in males and ≤60 in females). Controls undergoing catheterisation without angiographic CAD and no MI who were >45 years old
SNPs on chromosome 5p15.3 associated with myocardial infarction in Japanese population	21107343	Aoki A. et al.	J Hum Genet	2011	194 Japanese ancestry cases, 1539 Japanese ancestry controls	Cases with MI. Controls without MI and other diseases including asthma, breast cancer, lung cancer, hyperthyroidism, osteoporosis, chronic obstructive pulmonary disease, pollinosis and atopic dermatitis
A genome-wide association study identifies PLCL2 and AP3D1-DOT1L-SF3A2 as new susceptibility loci for myocardial infarction in Japanese	24916648	Hirokawa M. et al.	Eur J Hum Genet	2015	1666 Japanese ancestry cases, 3198 Japanese ancestry controls	Cases with MI having a coronary angiography and left ventricular asynergy on echocardiography. Controls without MI who were healthy volunteers and GWAS case samples for other diseases in the BioBank Japan without stable angina, unstable angina, or MI
Genome-Wide Association Study for Incident Myocardial Infarction and Coronary Heart Disease in Prospective Cohort Studies: The CHARGE Consortium	26950853	Dehghan A. et al.	PLoS One	2016	1570 European ancestry cases (at least), 21,618 European ancestry controls	Cases with MI (both fatal and non-fatal). Controls without MI and CHD
A genome-wide association study reveals susceptibility loci for myocardial infarction/coronary artery disease in Saudi Arabs	26708285	Wakil SM et al.	Atherosclerosis	2016	866 Saudi Arab ancestry cases, 371 Saudi Arab ancestry controls	Cases with unstable angina, non-ST or ST-elevation MI who underwent cardiac catheterization and were diagnosed through ECG manifestations and changes in biomarkers. Controls without MI and coronary stenosis who underwent surgery for heart valvular diseases
Genome-wide meta-analysis identifies novel susceptibility loci for myocardial infarction	33532862	Hartiala JA. et al.	Eur Heart J	2021	17,505 European ancestry cases, 454,212 European ancestry controls	Cases with: (1) Doctor-diagnosed MI; (2) International Classification of Diseases version-10 (ICD10) main and secondary; (3) Self-reported MI. Controls without MI
Uncovering myocardial infarction genetic signatures using GWAS exploration in Saudi and European cohorts	38072966	Al-Ali AK. et al.	Sci Rep	2023	3950 Saudi-Arabian cases, 2324 Saudi-Arabian controls	Cases with MI visiting the hospital age 5–66. Controls without MI and coronary stenosis who underwent surgery for heart valvular diseases

Table showing the characteristics of the nine GWAS of MI identifying genome-wide significant SNPs. Cases and controls definitions are reported as in the studies. GWAS, Genome-wide association studies; MI, Myocardial infarction [5,25,30,31,32,33,34,35,36].

## Data Availability

The original data presented in the study are openly available at https://www.ebi.ac.uk/gwas/home (1 October 2025).

## Supplementary Materials

The following supporting information can be downloaded at: https://www.mdpi.com/article/10.3390/jcdd13030127/s1, Table S1: GWAS significant SNPs associated with MI in European cohorts and their characteristics; Table S2: GWAS significant SNPs associated with MI in non-European cohorts and their characteristics.

## Abbreviations

The following abbreviations are used in this manuscript:

CAD	Coronary Artery Disease
CARDIoGRAM	Coronary Artery Disease Genome-Wide Replication and Meta-Analysis
C4D	Coronary Artery Disease Genetics Consortium
eQTL	Expression quantitative trait locus
FH	Familial hypercholesterolemia
GRACE	Global Registry of Acute Coronary Events
GRS	Genetic risk score
GWAS	Genome-wide association study
IL-1β	Interleukin 1-beta
IL-6	Interleukin-6
LD	Linkage disequilibrium
LDL	Low-density lipoprotein
LDLR	Low-density lipoprotein receptor
lncRNA	Long non-coding RNA
MI	Myocardial infarction
PCA	Principal component analysis
PSCK9	Proprotein convertase subtilisin/kexin type 9
PRS	Polygenic risk score
SNP	Single-nucleotide polymorphism
TIMI	Thrombolysis in Myocardial Infarction
TWAS	Transcriptome-wide association studies
